# Slowing down glioblastoma progression in mice by running or the anti-malarial drug dihydroartemisinin? Induction of oxidative stress in murine glioblastoma therapy

**DOI:** 10.18632/oncotarget.10723

**Published:** 2016-07-20

**Authors:** Dieter Lemke, Hans-Werner Pledl, Markus Zorn, Manfred Jugold, Ed Green, Jonas Blaes, Sarah Löw, Anne Hertenstein, Martina Ott, Felix Sahm, Ann-Catherine Steffen, Markus Weiler, Frank Winkler, Michael Platten, Zhen Dong, Wolfgang Wick

**Affiliations:** ^1^ German Cancer Consortium (DKTK), German Cancer Research Center (DKFZ), Heidelberg, Germany; ^2^ Clinical Cooperation Unit Neurooncology, German Cancer Research Center (DKFZ), Heidelberg, Germany; ^3^ Neurology Clinic and National Center for Tumor Diseases, University of Heidelberg, German Cancer Research Center (DKFZ), Heidelberg, Germany; ^4^ Central Laboratory of Heidelberg University Hospital, German Cancer Research Center (DKFZ), Heidelberg, Germany; ^5^ Core Facility Small Animal Imaging Center, German Cancer Research Center (DKFZ), Heidelberg, Germany; ^6^ Clinical Cooperation Unit Neuroimmunology and Brain Tumor Immunology, German Cancer Research Center (DKFZ), Heidelberg, Germany; ^7^ Department of Neuropathology, Institute of Pathology, University of Heidelberg, German Cancer Research Center (DKFZ), Heidelberg, Germany; ^8^ Clinical Cooperation Unit Neuropathology, German Cancer Research Center (DKFZ), Heidelberg, Germany; ^9^ Department of Neurosurgery, Tongji Hospital, Tongji Medical College, Huazhong University of Science and Technology, Wuhan, China

**Keywords:** dihydroartemisinin, glioblastoma, physical exercises (PE), therapy, physical exercise

## Abstract

Influencing cancer metabolism by lifestyle changes is an attractive strategy as - if effective - exercise-induced problems may be less severe than those induced by classical anti-cancer therapies. Pursuing this idea, clinical trials evaluated the benefit of e.g. different diets such as the ketogenic diet, intermittent caloric restriction and physical exercise (PE) in the primary and secondary prevention of different cancer types. PE proved to be beneficial in the context of breast and colon cancer.

Glioblastoma has a dismal prognosis, with an average overall survival of about one year despite maximal safe resection, concomitant radiochemotherapy with temozolomide followed by adjuvant temozolomide therapy. Here, we focused on the influence of PE as an isolated and adjuvant treatment in murine GB therapy.

PE did not reduce toxic side effects of chemotherapy in mice administered in a dose escalating scheme as shown before for starvation. Although regular treadmill training on its own had no obvious beneficial effects, its combination with temozolomide was beneficial in the treatment of glioblastoma-bearing mice. As PE might partly act through the induction of reactive oxygen species, dihydroartemisinin - an approved anti-malarial drug which induces oxidative stress in glioma cells - was further evaluated *in vitro* and *in vivo*. Dihydroartemisinin showed anti-glioma activity by promoting autophagy, reduced the clonogenic survival and proliferation capacity of glioma cells, and prolonged the survival of tumor bearing mice. Using the reactive oxygen species scavenger n-acetyl-cysteine these effects were in part reversible, suggesting that dihydroartemisinin partly acts through the generation of reactive oxygen species.

## INTRODUCTION

Cancer metabolism is an attractive target in tumors, especially those that are largely resistant against radio- and chemotherapy. As tumor cells rely more on anaerobic glycolysis compared with non-tumor cells, which is called the Warburg effect, glycolysis inhibitors were tested in cancer therapy [1]. Unfortunately, although this strategy has been pursued for many years and many different compounds are available now, there is only a very limited amount of clinical trials testing glycolysis inhibitors and, to our knowledge, no inhibitor has been approved for cancer therapy yet [2].

Similarly, life style factors which could influence cancer metabolism have for many years been the focus of epidemiologic studies [3–6], and indeed the influence of eating habits and physical exercise (PE) on cardiovascular morbidity and mortality is well known. Interest is growing in deciphering the impact of these factors on both the prevalence of different cancer types, as well as their significance in cancer therapy. PE has been shown to have beneficial effects in primary and secondary prevention of breast and colon cancer [7–11] and may also reduce cancer-related fatigue [12, 13].

Life style factors – such as caloric restriction and PE - may influence cancer therapy by several mechanisms, including changing the levels of growth factors (such as insulin-like growth factors), sex hormone profiles, reactive oxygen species (ROS), and inflammatory responses [14–18]. Pedersen et al. recently demonstrated that PE in mice on its own reduced tumor growth through epinephrine- and IL6 dependent NK cell redistribution into tumor tissue [19].

Interestingly, Rafaghello et al. showed that low-glucose or low-serum media protected primary glial cells, but not glioma and neuroblastoma cell lines against toxic levels of oxidative stress induced by hydrogen peroxide or cyclophosphamide chemotherapy. They translated their *in vitro* work into an *in vivo* model demonstrating that a starvation period of 48 h reduced toxicity of cyclophosphamide- and etoposide-chemotherapy in starved mice allowing the use of higher dosages of chemotherapy, which resulted in longer survival of neuroblastoma bearing mice. This process was named differential stress response (DSR), which they suggest occurs due to the lower resistance of tumor cells to altering levels of ROS compared with healthy cells [20]. Woolf et al. give an interesting overview about the metabolic changes induced by ketogenic diet which improve survival in animal models of malignant gliomas and can potentiate the anti-tumor effect of chemotherapies and radiation treatment [21]. Although, the ketogenic diet on its own did not show anti-glioma activity in a small clinical trial [22].

Here, we focused on the beneficial effects of PE as an isolated and adjuvant treatment strategy in glioblastoma (GB) therapy. GB has an average overall survival of less than 2 years despite maximal safe resection, concomitant radiochemotherapy with temozolomide (TMZ) followed by adjuvant TMZ-therapy [23].

We were interested to test whether PE is suitable to induce a DSR protecting non-tumor cells from TMZ induced toxicity and would thus allow applying higher doses of TMZ. We chose a forced exercise model with treadmill (TM) training over voluntary free-wheel activity. It has been demonstrated that this approach was more effective in inducing the molecular correlates of physical exercise, both in the muscle but also in the brain, where elevated levels of the mitochondrial complexes I and V as well as the anti-oxidative enzyme Mn-SOD were detected [24, 25].

Given that PE's mode of action might include induction of ROS, we sought to model this *in vitro* using the anti-malarial drug dihydroartemisinin (DHA), which has been shown to form ROS in leukemia cells and thereby induce anti-oxidative enzymes [26]. Artesunate which is metabolized to DHA was suggested to be used in the treatment for recurrent glioma in a cocktail with eight other drugs [27, 28].

Given the limited preclinical data about DHA, this approved anti-malarial drug might be a potentially interesting agent when combined with TMZ or radiotherapy in glioma therapy. It was demonstrated that DHA increased TMZ-toxicity in C6 rat glioma cells *in vitro* in a ROS-dependent mechanism [29]. Moreover, DHA is supposed to induce autophagy, to reduce invasivity of glioma cells and to target glioma stem cells [30–32]. DHA could therefore be an interesting agent in the treatment of GB sharing in part the mechanism of action with PE by inducing oxidative stress pharmacologically.

## RESULTS

### PE slows down tumor growth but does not induce a differential stress response

**Figure 1:**
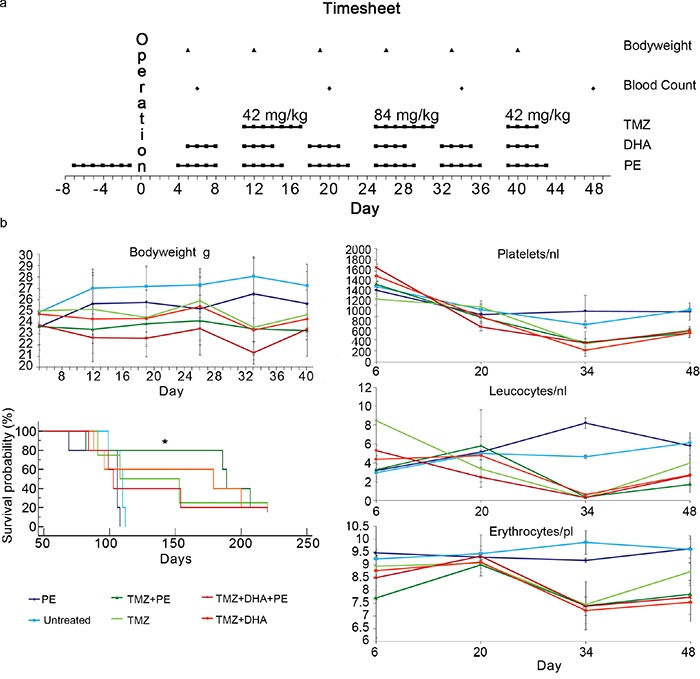
PE shows synergistic anti-tumor activity when combined with TMZ *in vivo* but does not protect against chemotherapy-induced toxicity **a.** Study protocol of the T269 *in vivo* experiment detailing the procedure timepoints. **b.** Kaplan-Meier survival plot as well as the course of blood counts and bodyweight are depicted for the T269 animal experiment. The time line corresponds to to days post operation. Only animals treated with PE combined with TMZ showed a significant benefit when compared with untreated animals (* p<0.05).

To test the effects of PE in the context of GB chemotherapy with TMZ, mice were habituated to daily moderate endurance training on a treadmill. Mice were trained at a speed of 18 meters per minute for 45 minutes at 5 of 7 days per week reflecting a moderate intensity which corresponds to 50-75% of the maximum oxygen consumption as shown before in a mastocytoma mouse model *in vivo* [33]. The treadmill and the incline as well as the detailed study protocol are illustrated in Figure 1a. TMZ was administered in a dose-intensified scheme of 7 days on and 7 days off for 2.5 cycles 10 days after the primary GB cell line T269 had been orthotopically implanted in nude mice. To address whether PE can induce a differential stress response with a higher resistance of the hematopoietic cells against TMZ-toxicity, the TMZ concentration was doubled in the second week of chemotherapy to 84 mg/kg.

DHA, known to induce oxidative stress, which could be one possible mechanism PE might act through, was given four days per week to imitate regular endurance training.

Blood counts and body weight were monitored to address hematopoietic toxicity and tolerance to treatment. Dose escalation of TMZ to 84 mg/kg showed toxic effects on the hematopoietic system with a severe reduction of the platelet and leucocyte counts in the TMZ-treated groups. PE and the administration of DHA did not induce a differential stress response with a reduced hematotoxicity. This was also true for body weight as a more general critical parameter for well-being. All animals treated at 84 mg/kg TMZ in the second week of chemotherapy suffered from loss of body weight, which did not occur in the untreated or PE-only-treated animals (Figure 1b).

**Figure 2:**
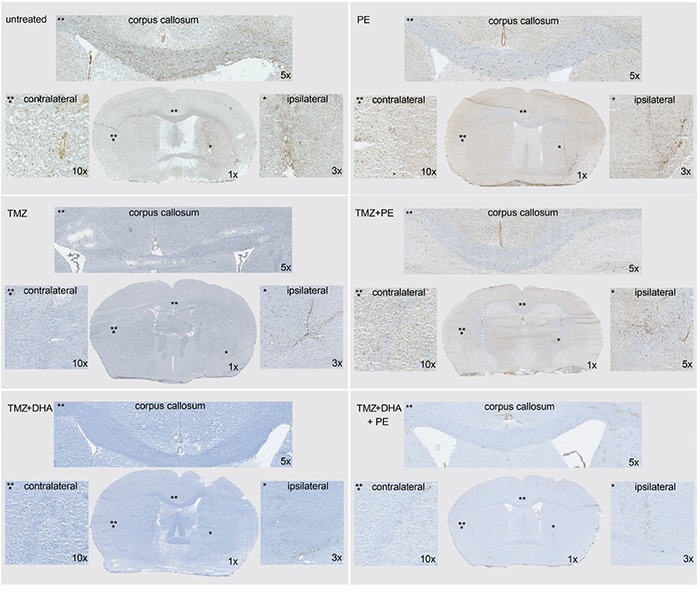
Combined treatment of DHA and/or PE with TMZ as well as TMZ-monotherapy show a massive reduction of invasiveness in orthotopically implanted T269 derived tumors in mice Tumor cells are stained with anti-human nestin antibody and appear in brown. The invasive pattern of the six treatment groups (control, PE only, TMZ, TMZ + PE, TMZ + DHA, TMZ + DHA + PE) are compared with the help of one overview image, and higher magnified images of the ipsilateral, corpus callosum- and contralateral regions.

Only PE combined with TMZ showed a significant survival benefit when compared with untreated animals in a pairwise analysis. The combined analysis of all the treatment subgroups did not show a significant treatment benefit in any subgroup with a p-value of 0.06 in the Kaplan-Meier log-rank test. As T269 primary glioma cells form very invasive tumors (Figure 2 upper raw), we were interested to see whether infiltration was reduced by the combination of TMZ and PE or DHA compared with single TMZ treatment. Untreated or only PE treated animals showed the largest tumors at the end of the treatment period which invaded into the contralateral hemisphere passing the corpus callosum (Figure 2 upper raw). After TMZ-monotherapy, invasion of T269 tumor cells was massively reduced without detectable tumor cells in the corpus callosum and the contralateral hemisphere which impeded the detection of a further anti-invasive effect in the combined treated mice (Figure 2 middle and lower raw).

To further evaluate the effects of PE on tumor growth, a second mouse experiment was conducted with the GB cell line LN-Z308, which allows monitoring of tumor growth with MRI because this cell line forms more bulky, non-invasive tumors. The TMZ dose was reduced to 21 mg/kg as LN-Z308 cells proved to be more sensitive *in vitro* compared with T269 cells (Supplementary Figure S1). Although T269 cells have an unmethylated MGMT-promoter status [34] suggesting that they might have a higher resistance against TMZ-treatment [35], they do not express MGMT protein and show an intermediate TMZ-sensitivity *in vitro* compared with the highly resistant MGMT-unmethylated T98G and the sensitive methylated LN-Z308 cells (Supplementary Figure S1).

**Figure 3:**
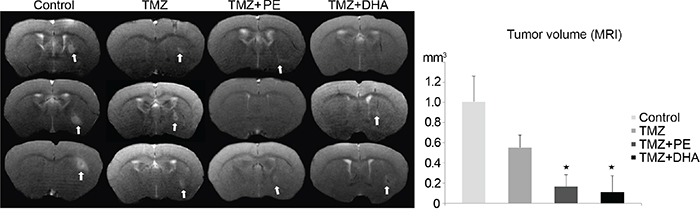
Combination therapy of either PE or DHA administered with TMZ is more effective than TMZ-monotherapy against LN-Z308 derived tumors in mice MR images and the statistical evaluation thereof demonstrate that *in vivo* the combined treatment of TMZ and either DHA or PE is more effective than TMZ alone in treating a LN-Z308 derived orthotopically xenotransplanted GB.

MRI, which was performed during the last chemotherapy week with TMZ, demonstrated that the addition of PE or DHA to TMZ resulted in smaller tumors compared with untreated or TMZ-only-treated animals. TMZ alone reduced tumor size to ^~^ 55% while the additional use of DHA or PE resulted in a tumor size of less than 20% compared with untreated animals (Figure 3).

### Effects of DHA on human glioma cells *in vitro*

**Figure 4:**
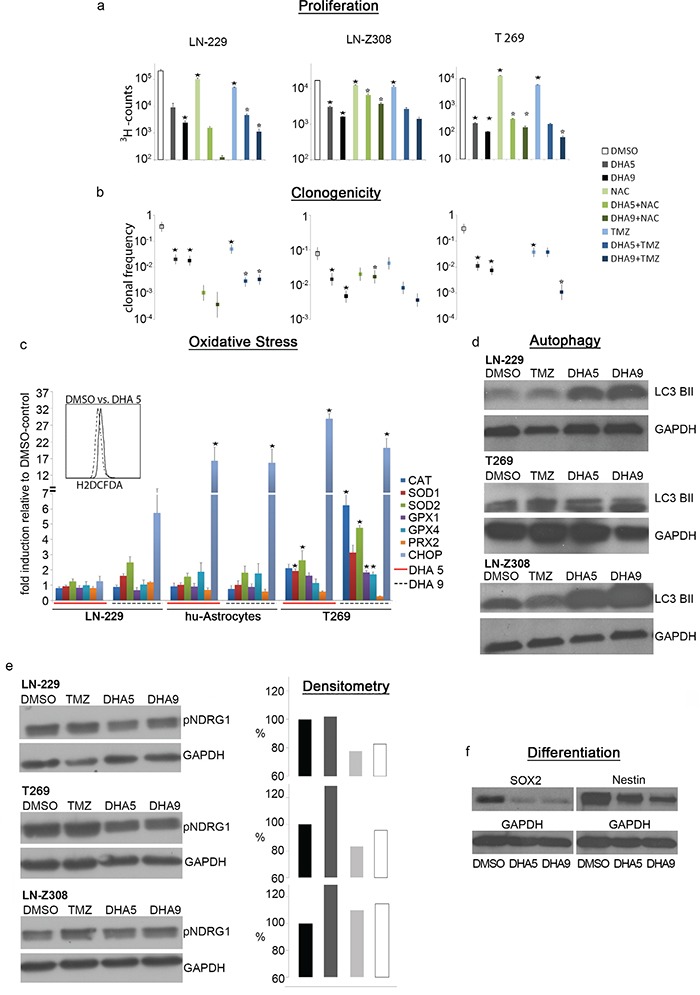
DHA exerts ROS-dependent anti-tumor activity **a.** DHA treatment is anti-proliferative measured by 3H-thymidine uptake, and anti-clonogenic, as measured by limiting dilution assay. **b.** Both effects are partially reversible by administration of the ROS-scavenger NAC at 2 mM concentration in LN-229 and T269 cells and 5 mM in LN-Z308 cells. Co-treatment of DHA and TMZ has synergistic effects in LN-229 and T269 cells. (* signifies a p-value <0.05 when comparing treated samples with DMSO-control, * signifies a p-value <0.05 when comparing DHA-treated samples alone with DHA+NAC- or DHA+TMZ-treated samples). **c.** DHA 5 mM induces oxidative stress in LN-229 cells measured by H2DCFDA in flow cytometry. DHA 5 and 9 mM can induce the upregulation of the mRNA levels of diverse anti-oxidative enzymes as well as CHOP. (* signifies a p-value <0.05 when comparing treated samples with DMSO-control). **d.** The combination of DHA 5 mM and 9 mM with TMZ induces autophagy measured after 72 h with LC3B II western blot in LN-229, T269 and LN-Z308 cells. **e.** TMZ treatment induces the expression of p-NDRG1 in T269 and LN-Z308 cells. P-NDRG1 levels are reduced when TMZ is combined with DHA 5 and 9 mM treatment. **f.** DHA reduces the expression of the stem cell markers Sox2 and Nestin in T269 cells as shown on western blot.

PE may act in many different ways on tumor tissue such as e.g. altering levels of blood glucose, ketone bodies, sex hormones and insulin, as well as stimulating the immune system [14–19]. As this complexity cannot be modelled *in vitro* we chose to further examine the role of ROS induction by PE. We chose DHA as a chemical compound to induce oxidative stress as this drug could, if effective, be directly used in the clinical setting. We used DHA in the dose range from 5-9 μM [36]. DHA caused a dose-dependent reduction of the proliferation rate in the glioma cell lines LN-229 and LN-Z308 as well as in primary T269 glioma cells (Figure 4a) measured by ^3^H-thymidine uptake. The use of the ROS scavenger N-acetyl-cysteine (NAC) at 5 mM could partially rescue the proliferation capacity in LN-Z308 cells. In LN-229 cells NAC, even when used with a lower concentration of 2 mM, showed additional toxicity when combined with DHA, while NAC at 2 mM had only a moderate rescuing effect in T269 cells treated with DHA. TMZ used at a concentration of 10 μM had a significant anti-proliferative effect on all the cells tested, although, after 72 h, this effect was a lot weaker compared with DHA monotherapy. The combination of TMZ and DHA at 9 μM proved to be more anti-proliferative in LN-229 and T269 cells compared with the monotherapies (Figure 4a).

We next evaluated the anti-clonogenic potential of DHA in limiting dilation assays of LN-229, LN-Z308 and T269 cells. Here again, DHA exerted a dose dependent effect by reducing the clonogenic potential between 17x in LN-Z308 cells to 40x in T269 cells at a concentration of 9 μM. Due to the additional toxicity of the ROS scavenger NAC even at a lower concentration of 2 mM, this radical scavenger could not rescue the DHA effects in LN-229 and T269 cells. In LN-Z308 cells, NAC at 5 mM could partly antagonize the anti-clonogenic effect of DHA used at the higher 9μM-dose illustrating that DHA acts in part *via* the induction of oxidative stress. A single treatment with TMZ at 10 mM was less anti-clonogenic than even the lower dose of DHA monotherapy.

The combination of DHA at 9μM and TMZ 10μM was more anti-clonogenic in LN-229 and T269 cells compared with the monotherapies (Figure 4b) arguing for a synergistic therapeutic effect.

As the rescue-experiments with NAC could only give a hint that ROS play a role in the mode of action of DHA we evaluated the mRNA-levels of enzymes antagonizing products of oxidative stress. The mRNA-expression of catalase (CAT), superoxide dismutase (SOD) 1 and 2, glutathione peroxidase (GPX) 1 and 4 as well as pyridoxine reductase 2 (PRX) were measured by qPCR in LN-229 cells, human astrocytes and T269. As a general marker of cellular stress we measured C/EBP homologous protein (CHOP) mRNA in qPCR and performed flow cytometric analysis with the fluorescent oxidative stress marker H_2_DCFDA in LN-229 cells after treatment with 5μM DHA.

H2DCFDA showed an increased fluorescence after treatment with DHA at 5 μM (Figure 4c). DHA at 9 μM induced cellular stress measured by a ~6-30x induction of CHOP in the tested cells.

Anti-oxidative enzymes were up-regulated on the mRNA-level for more than twofold exclusively in T269 primary glioma cells but not in human astrocytes or LN-229 cells. In detail, T269 cells showed upregulation of CAT, GPX1+4 and SOD2 (Figure 4c) after treatment with DHA at 9 μM.

Moreover, as DHA is supposed to act *via* autophagy [37], we addressed if it could induce autophagy in LN-229, T269 and LN-Z308 cells by measuring the protein levels of the early marker of autophagy, LC3 BII, in western blot (Figure 4d). Here, DHA was able to induce autophagy in all the cell lines tested as assessed by the higher levels of LC3 BII.

We further addressed phospho-p90RSK, phospho-Akt, phospho-p44/42 MAPK (Erk1/2) and phospho-S6 ribosomal protein levels in TMZ only or in TMZ and DHA treated cells to analyze whether these oncogenic pathways, which are in part responsible for resistance against TMZ therapy [38], are differentially induced by the combined *versus* the single agent treatment. DHA and TMZ did not reduce any of the above-mentioned phospho-protein levels compared with TMZ monotherapy. On the contrary, at least one phospho-protein was more activated in the different cell lines tested when comparing the combined therapy with the TMZ-monotherapy. TMZ monotherapy did not show an activation of the examined pathways compared with control treatment in these short lasting 72 hour *in vitro* experiments (Supplementary Figure S2).

Interestingly, the protein level of phospho-NDRG1, another gene responsible for TMZ-resistance [39], which was upregulated in all the cell lines tested after a single course of TMZ-chemotherapy, showed a reduction in the combined DHA and TMZ treatment compared with TMZ-monotherapy (Figure 4e).

Finally, we examined the protein levels of the stem cell markers sox2 and nestin in the primary glioblastoma cell line T269 after DHA treatment to assess whether DHA can induce differentiation. Both markers were clearly reduced after DHA treatment (Figure 4f).

## DISCUSSION

It has been observed that PE has positive effects in the primary and secondary prevention of breast and colon cancer, and that exercise is a strong independent predictor of survival in malignant recurrent glioma [5]. Clinical trials demonstrate that PE is feasible for heavily treated cancer patients and the adherence to PE e.g. in cancer patients receiving allogeneic stem cell transplantation was with 66% (inpatients) to nearly 80% (outpatients) high [40, 41]. We therefore set out to assess whether the addition of PE to standard TMZ treatment regime prolongs survival of glioblastoma bearing mice. We chose an orthotopic xenotransplanted tumor model of GB derived from the primary glioma-initiating cell-line (GIC) T269 [42]. GIC-lines have two significant advantages when modelling tumors: they better reflect the invasive growth pattern of human gliomas than do tumors derived from GB cell lines cultured in serum containing medium [43], and they may also be enriched in cancer stem cells which are thought to be responsible for therapy resistance and recurrence [44].

We found that while moderate PE by itself did not influence the survival of tumor-bearing CD1 nu/nu mice, in combination with TMZ it appeared to prolong the survival of mice. Although this only reached statistical significance when comparing TMZ+PE mice to untreated animals, we feel this is a particularly robust result given the small group sizes in this experiment (exacerbated by drop outs during repeated oral gavage and training). This is reinforced by the observation that the TMZ monotherapy group (the current standard therapy for GB) was itself not significantly different from the untreated group. Furthermore, differences between the treatment groups might have been masked by a very strong TMZ effect in some animals, which required the termination of the experiment after 220 days (Figure 1b).

This strong effect of TMZ might be explained by the dosing strategy, in that we utilized the dosing scheme established before in other mouse experiments [45, 46], in a dose-dense regimen (7 days on/7 days off) resulting in a cumulative TMZ dose of ~ 1g/kg over 3 weeks. During the escalated dose phase this was sufficient to test whether PE might induce a DSR, as mice clearly demonstrated signs of toxicity (Figure 1). However, the 1g/kg total dose would, in an average person weighing 75 kg and 180 cm tall, equate to a total dose of 75 g – far above the ~18 g a patient would receive over a standard 6 month therapy (6 cycles over 6 months with a daily TMZ dose of 200 mg/m^2^ body surface on 5 of 28 days per cycle, including the concomitant treatment during irradiation). Although one must demonstrate caution when making inferences between data from mouse models and the human situation, given that we utilized human GB cells, the amount TMZ might have been too high to detect any additional benefits of its combination with PE or DHA (in addition to the limitations of group size discussed above). This would also hold true when analyzing invasiveness of T269 derived tumors, where again the efficacy of the TMZ monotherapy in causing a massive decline of invasiveness (Figure 2) precluded the identification of any additional anti-invasive effects of PE and/or DHA.

We sought to determine if PE might induce an acute DSR, in much the same way as has been demonstrated for starvation [20], where non-tumor cells become resistant to otherwise toxic doses of chemotherapy. Such a mechanism would allow the use of higher doses of chemotherapy. However we found that all groups receiving TMZ at the escalated dose of 84 mg/kg bodyweight clearly demonstrated severe signs of intoxication with a loss of bodyweight and marked pancytopenia. This was by itself an unexpected result given previous publications suggesting that nude mice can tolerate up to 300 mg/m^2^ of TMZ, which corresponds to 100 mg/kg [47]. Intriguingly, the drop in body weight following TMZ therapy was less pronounced in the group with PE (Figure 1).

Given the difficulties of resolving any combinatorial effects of TMZ and PE or DHA in the first mouse experiment, we performed a further experiment utilizing LN-Z308 cells, which form non-invasive bulky tumors which can be analyzed using contrast-enhanced-MRI to compare treatment efficacy. We first performed a preliminary experiment (Supplementary Figure S1) to determine the relative sensitivity of LN-Z308 cells towards TMZ compared with T269 cells; although neither line expresses MGMT at the protein level (a key resistance factor to TMZ treatment) [35], LN-Z308 are about 4 times as sensitive as T269 (as shown by comparing TMZ dosages required to induce a similar effect on G2-arrest). We therefore lowered the dosage of TMZ to 21 mg/kg to maintain a similar effective toxicity for the tumor cells.

Encouragingly, this model did show significantly smaller tumors after a combination treatment of TMZ and PE or DHA when compared with TMZ monotherapy (Figure 3). For this second experiment we did not perform the TMZ+DHA+PE regimen as this proved to be less effective than TMZ combined with PE or DHA in the Kaplan-Meier analysis of the first animal experiment, probably due to accumulated toxicity.

We hypothesized that PE might mediate its effects in part through the induction of oxidative stress. As PE cannot be reproduced *in vitro*, we mimicked its potential effects pharmacologically using DHA, a known inducer of oxidative stress [29, 48, 49]. We confirmed that DHA treatment resulted in up-regulation of the mRNA-levels of anti-oxidative enzymes, especially in the primary T269 GICs (Figure 4b). Furthermore, preclinical *in vitro* data exist demonstrating various anti-glioma effects of DHA, with evidence suggesting that DHA treatment can also induce autophagy, exert an anti-invasive effect, and target stem cells in glioma [30–32]. Xu et al. showed that more than 80 proteins linked to the cytotoxicity of DHA in prostate cancer cells are differentially expressed in proteomic analysis including aminoacyl-tRNA biosynthesis and metabolic pathways as well as heat shock protein HSP70 underlining the complex mode of action of this agent.

Using clinically relevant doses of DHA we confirmed a dose dependent induction of the early autophagy marker LC3 BII (Figure 4d) in both the glioma lines LN-229 and LN-Z308, as well as the on primary glioma line T269.

We then tested the anti-glioma properties of DHA in our cell models, and observed a dose dependent anti-clonogenic and anti-proliferative effect in all three lines. These effects were in part reversible by the administration of the ROS-scavenger compound NAC, implying DHA might act through a ROS-dependent mechanism. The combination of DHA with TMZ showed synergistic anti-clonogenic and anti-proliferative action, confirming similar observations obtained using C6-rat glioma cells *in vitro* [29]. Many conflicting data exist on the induction of authophagy to sensitize glioma cells to TMZ showing that probably mild autophagy will enhance resistance while a stronger induction will lead to a higher sensitivity to TMZ [50, 51]. As our data showed a very robust induction of autophagy in the combined treatment of DHA and TMZ as well as synergistic anti-glioma activity, we believe that autophagy induced by DHA is beneficial in the treatment of GB. However, the definite role of autophagy in the combined treatment of TMZ and DHA would only be deciphered by blocking autophagy e.g. by silencing components of the autophagy pathway.

As recent studies suggest that TMZ can significantly increase driver mutations in recurrent glioma, particularly in the retinoblastoma and Akt-mTOR pathways [38], we examined the activity of these pathways by measuring the phospho-protein levels of p90RSK, Akt, p44/42 MAPK (Erk1/2) and S6 ribosomal protein. As the combined treatment of DHA and TMZ did not reduce the activity of the afore mentioned pathways in 72 hour *in vitro* experiments, this is unlikely to represent DHA's therapeutic mechanism in our *in vitro* experiments (Supplementary Figure S2).

Instead, we observed that protein levels of phospho-NDRG1, which rise after TMZ treatment and predict resistance against TMZ [39], were decreased by combining DHA and TMZ treatment (Figure 4e). To conclude, the inhibition of p-NDRG1 by DHA as well as DHA-induced ROS represent two possible mechansims how DHA might overcome therapy-induced resistance in gliomas [52]. Moreover, DHA exerted a differentiating effect on T269 cells leading to a down-regulation of the stem cell markers Sox2 and nestin which argues again for its use in the combination with TMZ to better target glioma stem cells.

To summarize, we could show that PE has an additive effect when combined with TMZ in the treatment of GB-bearing mice. Our escalating dosing scheme showed that PE does not mediate a DSR as shown before for starvation.

Given that many patients have difficulty in performing PE during treatment, we focused our efforts on mimicking the PE mediated induction of ROS [24, 25] using the anti-malarial drug DHA. DHA showed anti-glioma effects *in vivo* in mice. We were able to confirm previously reported anti-proliferative, anti-clonogenic and stem cell targeting effects of DHA in *in vitro* models (including two different glioma cell lines and primary T269 GIC).

As DHA is an approved drug which additionally suppresses TMZ–induced activation of phospho-NDRG1, a known mechanism of resistance, our data builds on previous results to warrant initiating clinical trials combining TMZ with DHA in the treatment of glioma patients.

Finally, for those patients able to perform PE, we believe various PE training paradigms should be evaluated in mice to develop an ideal protocol (balancing high versus low intensity training, or endurance versus resistance training), possibly including a specialized diet. Such a protocol could be translated into clinical trials to improve the treatment of glioma patients with a minimum risk of negative side effects.

## MATERIALS AND METHODS

### Cell culture

The primary GB cell line T269 was established from freshly dissected GB tissue from adult patients after informed consent and cultured as described before [42, 53]. Cells were seeded in neural sphere cell medium (NSCM) containing Dulbecco's modified Eagle's medium: F12 medium enriched with B27 supplement, basic fibroblast growth factor (bFGF) (20 ng/ml), epidermal growth factor (EGF) (20 ng/mL), and leukemia inhibitory factor (20 ng/ml). To propagate cells in culture they were split mechanically. The human glioma cell lines LN-Z308, T98G and LN-229 (ATCC, Manassas, USA) were kept in complete medium composed of Dulbecco‘s modified Eagle Medium (DMEM, High glucose, 4,5 g/l, PAA Laboratories) supplemented with 10% FBS and 1% penicillin/streptomycin. Cells were treated with TMZ (Sigma-Aldrich) diluted in dimethylsulfoxide (DMSO), dihydoartemisinin (Biotrend chemicals; Switzerland) and N-acetyl cysteine (Sigma-Aldrich). Concentrations are detailed in the figures.

### Clonogenicity

Clonogenic capacity was assessed by limiting dilution assay (LDA) [54]. Shortly, cells were dissociated with accutase. Afterwards, 96-well microwell plates were plated with 300, 50, 8, and 1 cells in 0.2 mL of NSCM or complete medium. After 3 weeks, microwell plates were analyzed for wells showing clones and clonal frequency as well as the level of significance were calculated with L-Calc free online software (STEMCELL Technologies, Cologne, Germany). Stem cell frequency was expressed by 1 divided by the minimum amount of cells necessary to form a colony. To evaluate TMZ and DHA effects LDA was also performed during treatment with these drugs in the indicated doses. TMZ and n-acetyl cysteine were administered once at the day of plating, DHA was given three times, at the day of plating as well as after 24 and 48 hours.

### Quantitative real-time PCR (qRT-PCR)

Total RNA was extracted using a RNA purification system (Qiagen, Hilden, Germany) and treated with RNase-free DNase I to remove genomic DNA (Roche, Mannheim, Germany). cDNA was prepared from 5 μg of total RNA using the Superscript RNase H–Reverse Transcriptase (Invitrogen, Karlsruhe, Germany) and random hexamers (Sigma-Aldrich, Taufkirchen, Germany). For qRT-PCR, gene expression was measured in an ABI Prism 7000 Sequence Detection System (Applied Biosystems, Foster City, CA, USA) with SYBR Green Master Mix (Eurogentec, Cologne, Germany) and primers at optimized concentrations [42]. Primers (Sigma-Aldrich) were selected to span exon–exon junctions if possible. Standard curves were generated for each gene and the amplification was 90–100% efficient. Relative quantification of gene expression was determined by comparison of threshold values. All results were normalized to glyceraldehyde-3-phosphate dehydrogenase (GAPDH). The sequences for the housekeeping gene glyceraldehyde-3-phosphate dehydrogenase and the genes evaluated were as follows:

**Table:** 

CAT	forward: 5′---TTTCCCAGGAAGATCCTGAC---3′
	reverse: 5′---ACCTTGGTGAGATCGAATGG---3′
CHOP	forward: 5′---CATCACCACACCTGAAAGCA---3′
	reverse: 5′---TCAGCTGCCATCTCTGCA---3′
GAPDH	forward: 5′---CTCTCTGCTCCTCCTGTTCGAC---3′
	reverse: 5′---TGAGCGATGTGGCTCGGCT---3′
GPX1	forward: 5′---TTCCCGTGCAACCAGTTTG---3′
	reverse: 5′---TTCACCTCGCACTTCTCGAA---3′
GPX4	forward: 5′---TACGGACCCATGGAGGAG---3′
	reverse: 5′---CCACACACTTGTGGAGCTAGAA---3′
PRX2	forward: 5′---CAGACGAGCATGGGGAAG---3′
	reverse: 5′---ACGTTGGGCTTAATCGTGTC---3′
SOD1	forward: 5′---AGGGCATCATCAATTTCGAG---3′
	reverse: 5′---TGCCTCTCTTCATCCTTTGG---3′
SOD2	forward: 5′---GGAAGCCATCAAACGTGACT---3′
	reverse: 5′---CTGATTTGGACAAGCAGCAA---3′

### Immunoblot analysis

Cells were lysed in 50 mmol/L Tris-HCl (pH 8) containing 120 mmol/L NaCl, 5 mmol/L EDTA, 0.5% Nonidet P-40, 2 μg/mL aprotinin, 10 μg/mL leupeptin (Sigma-Aldrich), and 100 μg/mL phenylmethylsulfonylfluoride. Protein levels were analyzed by immunoblot using 30 μg of protein per lane with the respective antibodies in concentrations recommended by the manufacturer. The antibodies used were rabbit anti-Nestin(1:200; Chemicon, Temecula, CA, USA), goat anti-Sox2 (1:400; R&D-systems), rabbit Phospho-NDRG1 (1:5000; Cell Signaling), rabbit anti-LC3 B antibody (ab51520) (1:3000; Abcam, Cambridge, UK), and the PathScan^®^ Multiplex Western Cocktail I from Cell Signalling (Boston, MA, USA) was used to analyze the Phospho-p90RSK, Phospho-Akt, Phospho-p44/42 MAPK (Erk1/2) and Phospho-S6 Ribosomal Protein levels. Protein bands were visualized using horseradish peroxidase-coupled secondary antibodies (Sigma-Aldrich). Equal protein loading was ascertained by Ponceau S staining as well as GAPDH staining with goat anti-GAPDH antibody (1:5000; Linaris, Germany) [42]. Densitometry of immunoblots was performed with the help of ImageJ (National Institutes of Health) after scanning of the blots with the help of Photoshop software to create TIFF-files.

### Flow cytometry

Cells were analyzed in a BD-FACS Canto II flow cytometer, final data were processed with the help of FlowJo flow cytometry analysis software (Treestar) [42]. To access ROS levels cells were stained with H2DCFDA (Thermo Scientific, Waltham, USA) after a 72 h treatment course with DHA 5 μM or DMSO as control.

### Proliferation

LN-Z308 and LN-229 cells at 2500 cells/well and T269 at 5000 cells/well were dissociated and plated in quadruplicates in 96- well plates in 200 μl volumes of medium. TMZ and n-acetyl cysteine were administered once at the day of plating, DHA was given three times, at the day of plating as well as after 24 and 48 hours. After 72 h, cells were pulsed for 24 h with [methyl-3H]thymidine (0.5 lCi), harvested (Tomtec, Hamden, CT, USA), and incorporated radioactivity was determined in a liquid scintillation counter (Wallac, Turku, Finland) [55].

### Animal experiments

All animal work was performed in accordance with the German animal protection law (Approving institution: Regierungspraesidium; Karlsruhe; animal proposal: 35-9185.81/G-99/08 G). Tumors were induced injecting 1×10^5^ T269 or LN-Z308 cells suspended in phosphate buffered saline (PBS) orthotopically into the right striatum of 6–12 weeks old athymic female mice (CD1 nu/nu, Charles River, Sulzfeld, Germany) by a stereotactic procedure. In the first experiment with T269 cells 8 animals were implanted per group. Six were used for Kaplan-Meier analysis, 2 animals for histological examinations. In the second experiment with LN-Z308 cells, 4 animals were implanted per group, of which 3 were used for analysis of tumor size by MRI.

To reduce pain, animals were anesthetized with xylazine and ketamine. Neurological symptoms were assessed daily. Symptomatic animals were rapidly sacrificed and brains were snap-frozen in liquid nitrogen for further analysis. Cryostat transverse brain sections (8 μm) were stained with hematoxylin/eosin (H&E) or with anti-human Nestin antibody (Chemicon International, Billerica, USA) on a Ventana Benchmark Immunostainer (Ventana Medical Systems, Tucson, USA) and analyzed by AxioVision software (Carl Zeiss, Jena, Germany).

To evaluate the influence of PE and DHA alone and in combination with TMZ *in vivo*, DHA (0,9 mg/kg bodyweight) was administered subcutaneously after preparation of a DHA stock solution containing 2.25 mg DHA, 10 μl TWEEN 80, 1g NaCO_3_ and 20 ml distillated water for four days of every chemotherapy cycle as established for malaria therapy in rodents [56]. TMZ was administered in a 0.5% methylcellulose solution in 0.9% NaCl by oral gavage. PE was performed for 45 min on 5 days per week with a moderate intensity of up to 18 m/min with an incline of the treadmill of 5°. The first week of exercises speed was continuously increased until the animals were used to it. If animals were too exhausted due to therapy or tumor growth to keep up with the speed of 18 m/min it was lowered. The slowest speed mice ran was 16 m/min for 40 min. The details of the first mouse experiment with T269 cells are provided in Figure 2a. In the second experiment with LN-Z308 cells all TMZ cycles were administered at 21 mg/kg bodyweight. This dose was systematically established as shown in Supplementary Figure S1.

Blood samples for the measurement of chemotherapy toxicity were taken from the submandibular vein, a maximum of 140 μl/25g bodyweight were collected. Tumor volumes were determined *via* T1-weighted magnetic resonance imaging (MRI) after intraperitoneal injection of gadolinium-DTPA. MRI was performed at day 40 of the LN-Z308 experiment during the last week of TMZ administration.

### Statistical analysis

Statistical significance was assessed by Student's t-test (Excel, Microsoft, Seattle, WA, USA) at p < 0.05 (significant). In animal experiments, Kaplan-Meier-analysis log rank test (SPSS-statistics, IBM) was used.

## SUPPLEMENTARY MATERIALS FIGURES



## Abbreviations

bFGF: Basic fibroblast growth factor
ERK: extracellular signal–regulated kinase
CAT: Catalase
CHOP: C/EBP homologous protein
DSR: differential stress response
DHA: dihydroartemisinin
DMSO: Dimethylsulfoxide
DSR: differential stress response
EGF: epidermal growth factor
FBS: fetal bovine serum
GIC: glioma initiating cells
GB: glioblastoma
GPX1 and 4: glutathione peroxidase
GAPDH: glyceraldehyde-3-phosphate dehydrogenase
H&E: Hematoxylin/Eosin
LDA: limiting dilution assay
MAPK: mitogen-activated protein kinase
MGMT: O6-Methylguanin-DNA-Methyltransferase
MRI: magnetic resonance imaging
NAC: N-acetyl-cysteine
NDRG-1: N-myc downstream regulated gene
NSCM: neural sphere cell medium
PRX2: Peroxiredoxin
PE: physical exercises
PBS: phosphate-buffered saline
ROS: reactive oxygen species
SOD1 and 2: superoxide dismutase
Mn: Manganese
TMZ: Temozolomide
