# Supplementary material for: Slowing down glioblastoma progression in mice by running or the anti-malarial drug dihydroartemisinin? Induction of oxidative stress in murine glioblastoma therapy
Source: Oncotarget. 2016 Jul 20;7(35):56713–25. doi: 10.18632/oncotarget.10723 (PMC5302947; doi:10.18632/oncotarget.10723)
Supplement: Supplementary file 1 [file oncotarget-07-56713-s001.pdf]

Slowing down glioblastoma progression in mice by running or the anti-malarial drug dihydroartemisinin? Induction of oxidative stress in murine glioblastoma therapy

SUPPLEMENTARY FIGURES

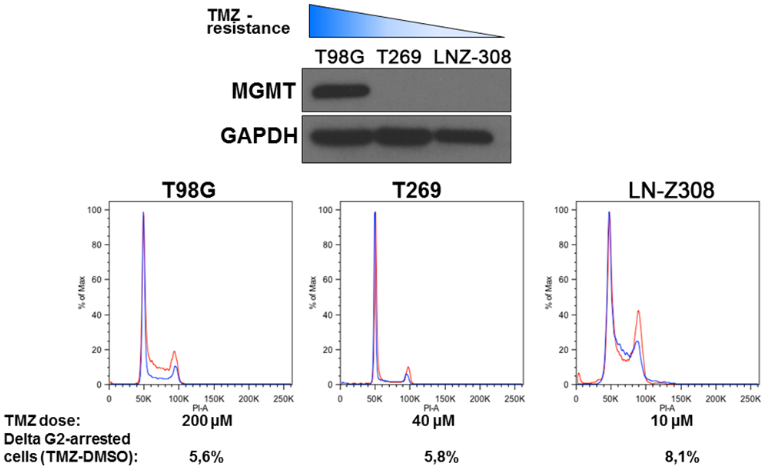

Supplementary Figure S1: Differential TMZ-sensitivity of glioma cells measured by G2-arrest. TMZ-sensitivity was evaluated in the primary MGMT unmethylated GB cell T269 as well as the GB cell lines T98G (unmethylated) and LN-Z308 (methylated) by G2-arrest with cell cycle analysis. MGMT is key resistance factor to TMZ treatment. MGMT-expressing T98G cells need TMZ at a dose of 200 µM to induce a G2-arrest of 5-6% while T269 and LN-Z308 cells need TMZ at 40 µM and 10 µM, respectively, to induce a comparable result.

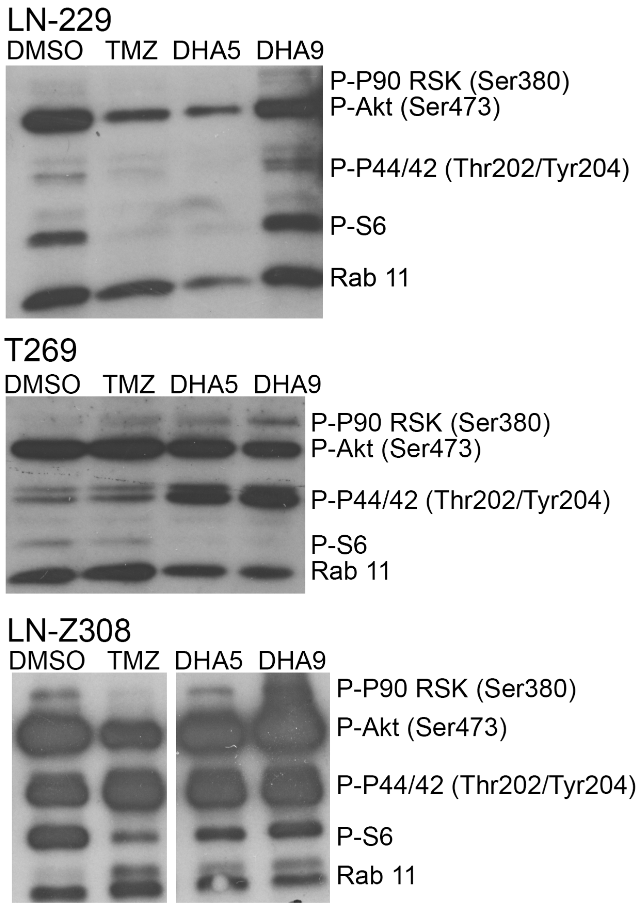

Supplementary Figure S2: DHA does not suppress the activation of Akt when combined with TMZ treatment. PathScan® Multiplex Western Cocktail was used to analyze the Phospho-p90RSK, Phospho-Akt, Phospho-p44/42 MAPK (Erk1/2) and Phospho-S6 Ribosomal Protein levels in LN-229, T269 and LN-Z308 cells. Rab 11 was used as loading control. TMZ alone does not induce while the addition of DHA is not able to inhibit the above specified phospho-proteins.
